# A high throughput screening system of coils for ELF magnetic fields experiments: proof of concept on the proliferation of cancer cell lines

**DOI:** 10.1186/s12885-019-5376-z

**Published:** 2019-02-28

**Authors:** Leonardo Makinistian, Eva Marková, Igor Belyaev

**Affiliations:** 1 Department of Radiobiology, Cancer Research Institute, Biomedical Center, Slovak Academy of Sciences, Dúbravská cesta 9, 845 05 Bratislava, Slovakia; 2 Department of Physics and Instituto de Física Aplicada (INFAP), Universidad Nacional de San Luis-CONICET, Ejército de los Andes 950, CP5700 San Luis, Argentina

**Keywords:** ELF magnetic fields, Cancer cell line proliferation, High throughput screening, U251, MDA-MB-231

## Abstract

**Background:**

It has been demonstrated that relatively small variations of the parameters of exposure to extremely low frequency magnetic fields (ELF-MF) can change significantly the outcome of experiments. Hence, either in trying to elucidate if these fields are carcinogenic, or in exploring their possible therapeutic use, it is desirable to screen through as many different exposures as possible. The purpose of this work is to provide a proof of concept of how a recently reported system of coils allows testing different field exposures, in a single experiment.

**Methods:**

Using a novel exposure system, we subjected a glioblastoma cancer cell line (U251) to three different time modulations of an ELF-MF at 60 different combinations of the alternated current (AC) and direct current (DC) components of the field. One of those three time modulations was also tested on another cell line, MDA-MB-231 (breast cancer). After exposure, proliferation was assessed by colorimetric assays.

**Results:**

For the U251 cells, a total of 180 different exposures were tested in three different experiments. Depending on exposure modulation and AC field intensity (but, remarkably, not on DC intensity), we found the three possible outcomes: increase (14.3% above control, *p* < 0.01), decrease (16.6% below control, *p* < 0.001), and also no-effect on proliferation with respect to control. Only the time modulation that inhibited proliferation of U251 was also tested on MDA-MB-231 cells which, in contrast, showed no alteration of their proliferation on any of the 60 AC/DC field combinations tested.

**Conclusions:**

We demonstrated, for the first time, the use of a novel system of coils for magnetobiology research, which allowed us to find that differences of only a few μT resulted in statistically different results. Not only does our study demonstrate the relevance of the time modulation and the importance of finely sweeping through the AC and DC amplitudes, but also, and most importantly, provides a proof of concept of a system that sensibly reduces the time and costs of screening.

**Electronic supplementary material:**

The online version of this article (10.1186/s12885-019-5376-z) contains supplementary material, which is available to authorized users.

## Background

The relation between extremely low frequency magnetic fields (ELF-MF) and cancer has been object of scientific, but also public discussions for decades, forcing international organizations to release thorough communications on the subject [1–3]. In particular, the possibility of a causal link between childhood leukemia and ELF-MF has raised major concern in the public opinion, giving place to several epidemiological studies [4–6].

These fields, however, are also of scientific interest for the opposite reason: the possibility of their therapeutic use [7–11]. Indeed, there are several in vitro experiments reporting inhibition of cancer cell proliferation [12–16], and also some in vivo studies point in the same direction: significant reduction of tumor growth has been reported in mice with induced tumors of breast cancer [17, 18], sarcoma [19], melanoma [20–22], and Ehrlich ascites carcinoma [23].

It is worth noting that the majority of the studies on the effect of ELF-MF reported only a single exposure, i.e., one single set of parameters of MFs is usually evaluated. These single-exposure studies could be appropriate for the purpose of, e.g., identifying atomic, molecular, or supramolecular targets of the fields (such as ions, molecules, ion channels, the membrane as a whole, etc.), or understanding the downstream events after transduction. Also, simply by showing that fields with intensity lower than a supposedly safe threshold are actually effective to elicit a potentially harmful response, safety of the said threshold can be solidly challenged. In contrast, when the aim of a study is to explore the possibility of a therapeutic use of ELF-MF, the need for testing many different sets of parameters, i.e., screening within some range of the different variables that define the exposure, is desirable. Indeed, screening is a key approach in exploration of new, and optimization of known treatments (drugs being the paradigmatic example). The appeal for screening is further supported by the dependence of ELF-MF effects on many physical and biological variables such as MF alternating current (AC) frequency, waveform, time modulation, AC to direct current (DC) field intensity ratio, genotype, physiological state, cell density, temperature, and concentrations of ions and radicals [24–31]. The purpose of this article is to report experiments that, for the first time, provide a proof of concept of how a novel system of coils, described elsewhere [32], can be used for screening, in a single experiment, 60 different combinations of DC and AC intensities for a given time modulation of the MFs.

## Methods

### Exposure system and background fields

**Fig. 1:**
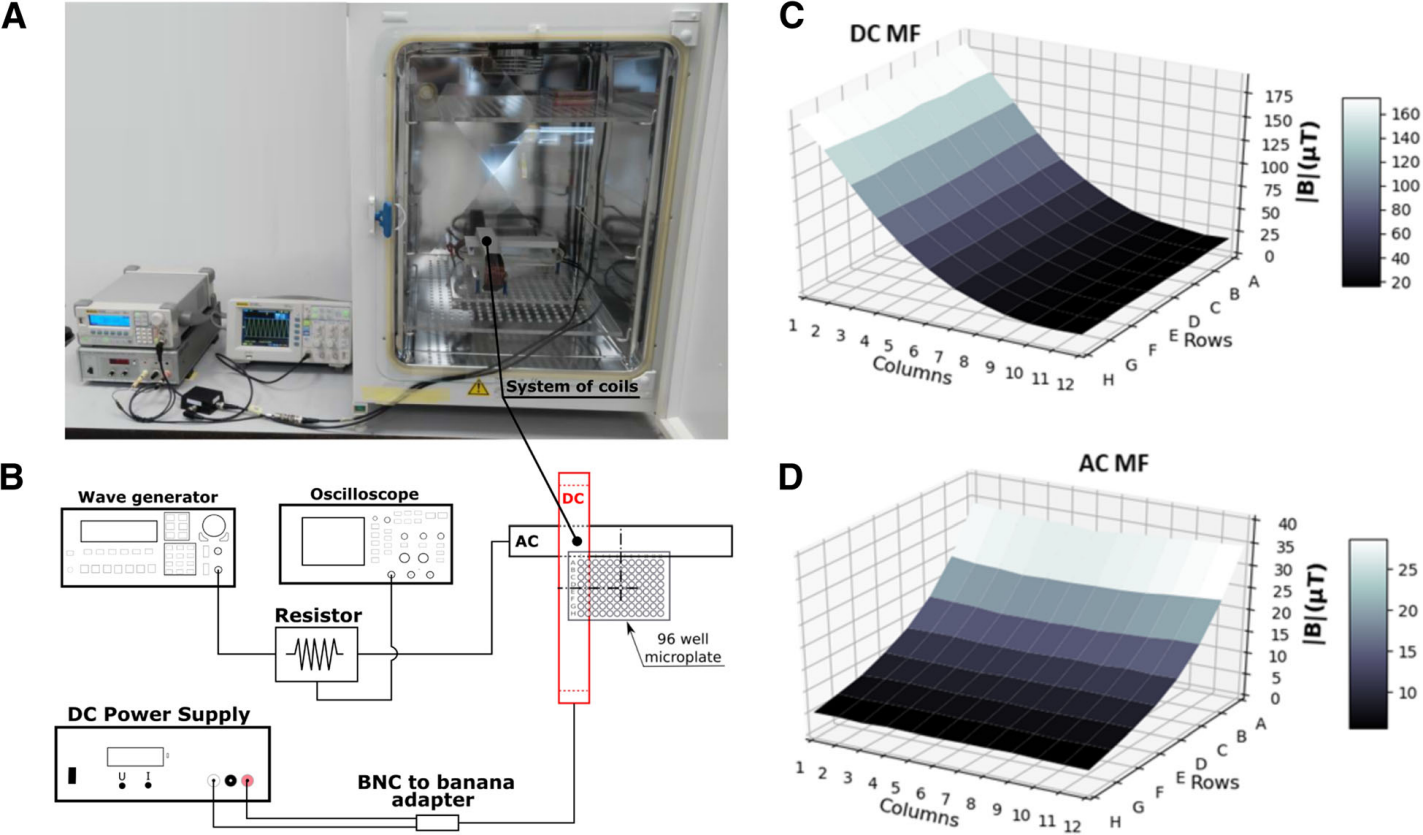
The exposure system and magnetic fields. **a** Photo, and (**b**) scheme of the exposure system. **c** DC MF (background plus coil-generated), and (**d**) AC MF, for the 96 wells of the exposed microplate (columns 1–12, rows A-H)

The system consisted of two flatten, orthogonal coils which produced non-homogeneous MFs and were described in detail elsewhere [32] (Fig. 1a-b). Briefly, they are oblong coils, fixed to a polymethyl methacrylate (PMMA) support at a right angle to each other. When energized, they produce a MF which is maximal in their proximity, and decreases with distance (Fig. 1c-d). Since a standard 96-well microplate for cell cultures is placed perpendicular/parallel to the coils, it turns out that one of the coils generates fields (mostly) parallel to the rows of the microplate, while the other generates them parallel to the columns. It is clearly seen that the DC MF has isolines parallel to the columns of the 96-well plate (Fig. 1c), while the AC MF has them parallel to the rows (Fig. 1d). As a result, and as a first approximation, all wells in the same row were exposed to the same AC field, while all the ones in the same column were exposed to the same DC field. The DC background field present in the incubator with both coils unenergized was thoroughly assessed [33] with an HCM5883L 3-axis magnetometer (Honeywell, New Jersey, NY) and its value at the center of each of the wells of the 96-well microplate was vector-added to the field generated by the DC coil in order to know the net DC field that the cultures were exposed to (background DC values were between 33.6 μT and 38.0 μT throughout the plate). Radiofrequency (RF) background level was of 0.5 mV/m inside the incubator, as measured with a TM-196 RF 3-axis field strength meter (Tenmars, Taiwan). The AC background field was measured with a TM-192 3-axis magnetometer (Tenmars, Taiwan) and was homogeneous within the region of both microplates (exposed and control): ~ 100 nT_rms_ with the incubator heating system off, and ~ 1 μT_rms_ with the heating system on. The DC current was delivered by a B5-45A DC Power Supply (Izmeritel, Russia) with a ripple of ~ 1%. The AC signal was generated by a DG1022 arbitrary wave generator (Rigol, Beaverton, OR).

The waveform was monitored with a DS1052E digital oscilloscope (Rigol, Beaverton, OR) hooked to a 50 Ω resistor in series with the AC coil (Fig. 1b). In a previous work [32], it was proven that Joule heating of the coils is acceptable (0.21 °C) upon the injection of a total current of 1.2 A to the system (600 mA to each coil). In the present work we injected 450 mA to the DC coil and 100 mA_peak_ (~ 71 mA_rms_ for a pure sine) to the AC coil; making the angle between DC and AC MF vectors range between 91° and 126°. Both DC and AC_peak_ currents were the same for all experiments. Since heating is proportional to the squared current, the Joule heating in the experiments reported here was more than 5 times lower than for 1.2 A, corresponding to a maximum temperature increase of ≲ 0.04 °C. Further, two signals with the same DC and AC amplitudes (but different carrier frequency, see below) elicited different effects on the same cell line, therefore, thermal load confounding can safely be ruled out in our experiments. The control microplate was placed in the upper, PMMA shelf of the incubator (Fig. 1a), and the control wells were exposed to an average DC background field of 41.7 μT (B_horiz_ = 27.7 μT, B_vert_ = − 31.2 μT) with a ~ 98% homogeneity, as measured with the incubator’s door closed. We also measured the fields at the site of the controls with the coils energized and unenergized (disconnected) and did not detect any difference. Hence, according to our instruments’ detection limits and typical variability of measurements, DC field at the control plate due to the DC coil (if any) was no greater than ~ 2 μT, while AC field due to the AC coil was no greater than ~ 100 nT (rms).

### Magnetic field modulations

**Fig. 2:**
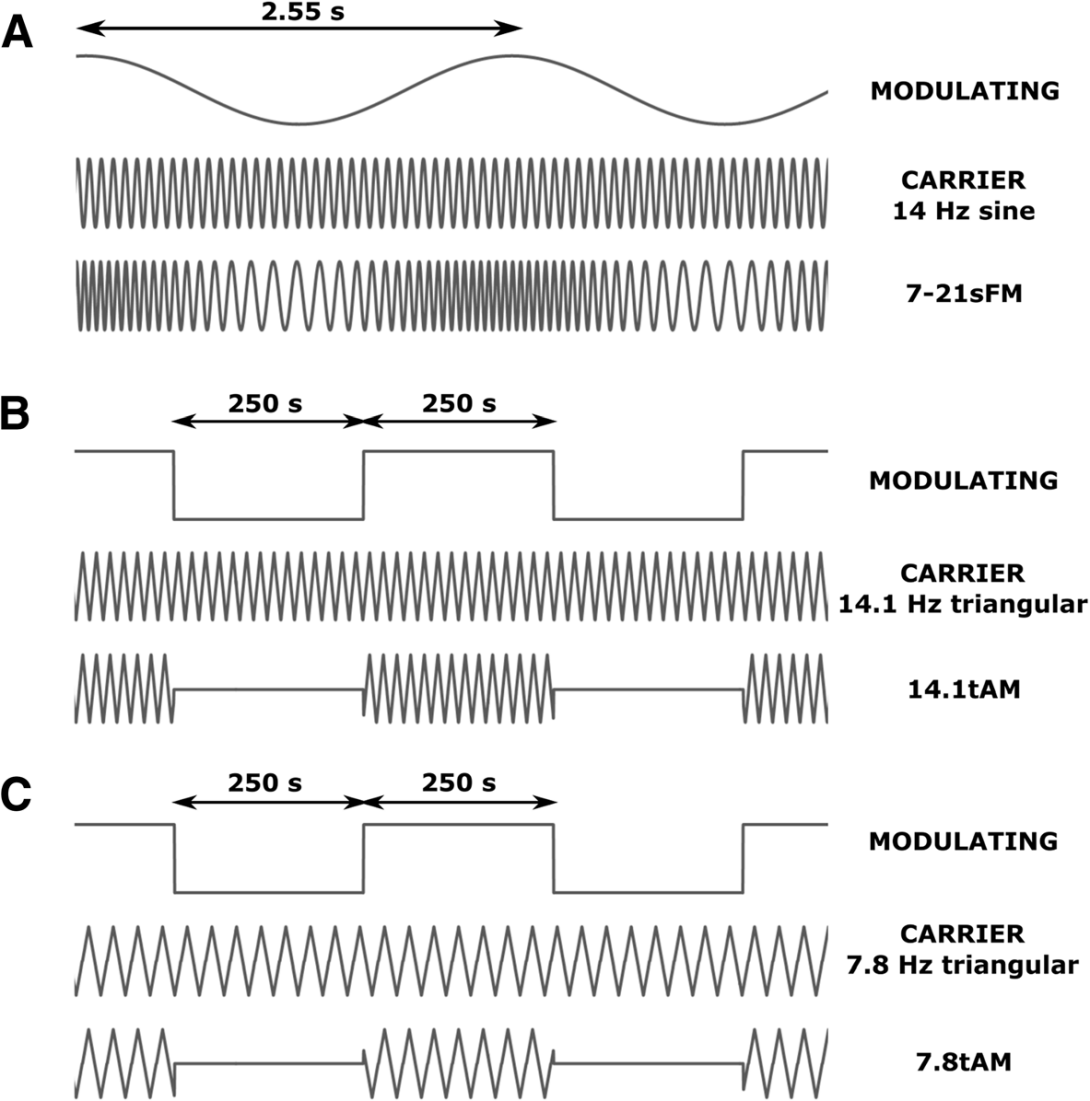
Signals’ details. **a** Signal 7-21sFM: frequency modulated sine wave, between 7 and 21 Hz, complete sinusoidal sweep in 2.55 s. **b** Signal 14.1tAM: 14.1 Hz triangular symmetric wave, amplitude modulated by a 2 mHz (500 s period) 50% duty cycle ^(*)^ square wave. **c** Signal 7.8tAM: same as 14.1tAM, only that the carrier frequency was 7.8 Hz. ^(*)^ During the “low” intervals of the modulating square wave (for both, 7.8tAM and 14.1tAM), the signal injected to the coil did not went strictly to zero; instead, it went down to 1% of its peak value. For all three signals and all experiments, peak intensity of the AC current was 100 mA_peak_, and the DC current was of 450 mA

We used Schumann frequencies in the design of our signals for two reasons: 1) these frequencies have been proposed to have an intimate bond with biological phenomena [34], and 2) to the best of our knowledge they have rarely been used in in vitro ELF-MF exposure experiments [35]. We tested three different waveforms: 1) Signal 7-21sFM (Fig. 2a) was a frequency modulated sine wave, between 7 and 21 Hz, with a complete sinusoidal sweep in 2.55 s (this duration was inspired by the experiments of Buckner et al. [36]). 2) Signal 14.1tAM (Fig. 2b) was a 14.1 Hz triangular symmetric wave, amplitude modulated by a 2 mHz (500 s period) 50% duty cycle square wave. 3) Signal 7.8tAM (Fig. 1c), was just as 14.1tAM, but with a carrier of 7.8 Hz (instead of 14.1 Hz). For these last two signals, it must be noted that during the “low” intervals of the modulating square wave, the current injected to the coil did not went strictly to zero. Instead, it went down to 1% of its peak value. The frequency range for 7-21sFM and the carriers of 14.1tAM and 7.8tAM were chosen considering the first three Schumann resonance frequencies: 7.8 Hz, 14.1 Hz, and 20.3 Hz [37, 38]. Sinusoids are, by far, the most tested signals, and hence, they were our first choice for the carrier. However, when we got no effect upon exposure to 7-21sFM (see below), we decided to change the carrier to triangular, and also to introduce the intermittence given by the square amplitude modulation. With regards to the 500-s period, while the exact value was rather arbitrary, the underlying rationale was that a continuous signal could trigger the onset of an adaptation mechanism making the cultures “ignore” the exposure. Upon observing an effect with 14.1tAM, we thought that it would be a sensible idea to change only one parameter (the carrier frequency) and leave the waveform (triangular) and the intermittence period fixed.

### Cells and proliferation assays

During the experiments cells were kept in a HERACell 150i CO_2_ Incubator (Thermo Fisher Scientific, MA) at 37 °C and 5% CO_2_. In preliminary experiments, we titrated the amount of seeded cells of both cell lines to achieve about 80% confluence after 72 h incubation. A first series of experiments was done exposing the U251 cell line - human glioblastoma, pleomorphic/astrocytoid, adherent cells from the ECACC-European Collection of Authenticated cell cultures, Operated by Public Health England (cat.num.09063001) - for 72 h continuously, starting immediately after seeding 1250 cell/well in 100 μl culture medium (DMEM, 2 mM glutamine, 10% FBS, 1% P-S) in a 96-well microplate until the end of exposure. After exposure, proliferation was assessed by adding 10 μl/well of 3-(4,5-dimethylthiazol-2-yl)-5-(3-carboxymethoxyphenyl)-2-(4-sulfophenyl)-2H-tetrazolium (MTS) reagent (Promega, WI), followed by a 3 h incubation before photometric assessment at 490 nm (with base at 690 nm) in an xMark Microplate Absorbance Spectrophotometer (BIO-RAD, CA). Two signals were tested: 14.1tAM and 7-21sFM. In a second series of experiments, we tested a third signal, 7.8tAM, on two cell lines: U251 (same as for the first series) and human epithelial breast cancer cells MDA-MB-231 from the ECACC-European Collection of Authenticated cell cultures, Operated by Public Health England (cat. Num. 92,020,424), seeding 3000 cells/well in 200 μl culture medium (DMEM, 2 mM glutamine, 10% FBS, 1% P-S) in 96-well microplate. After exposure, cells were incubated for 3 h with 50 μl of 1 mg/ml solution of 3-(4,5-dimethylthiazol-2-yl)-2,5-diphenyltetrazolium bromide (MTT). Then plates were centrifuged (1200 rpm for 5 min), the medium discarded, and 150 μl of DMSO added; after shaking the plates for 10 min, readings were done at 540 nm (with base at 690 nm). For both series and all experiments, two plates (“control” and “exposed”) were filled completely with a 12-channel pippete using the technique of reverse pippeting to enhance repeatability [39].

### Statistical analysis

The raw measurements of the MFs, for each of the 96 wells (Fig. 1c-d), underwent a two-step process. First, all edges were discarded. This was due to the commonly known possible evaporation of medium in the outer wells of multi-well plates. These wells were filled with medium with cells as all the others, but they were not analyzed, so only 60 out of the 96 wells were analyzed. Secondly, the exact values of the measurements (taken with a precision of 0.1 μT) were rounded up to integer values coinciding with the actual measurements within ±3 μT and ± 2 μT for the DC and AC MFs, respectively. This made possible to group together wells exposed to similar fields. Although seeded completely, only 8 wells of the control plate (wells D5 through E8) at a homogeneous DC MF of 41.7 μT were assayed and included in the statistical analysis. In each experiment, the absolute colorimetric readings of the exposed and also of the control wells were normalized (i.e., divided) by the average of the 8 control wells, hence the average control was always identical to 1 and the proliferation of the exposed wells are reported relative to control. After finding significant differences with a one-way ANOVA analysis, Dunnet’s test was used to compare exposed against control wells, and the Tukey HSD multiple comparison test was used to evaluate differences within the same plate, but for different AC MF intensities. All the statistical analysis was performed with the STATISTICA software on the spectrometric raw data (see Additional file 1: MagneticFields&ProliferationAssaysRawData.xlsx). 3D plots of relative proliferation were prepared with the Python Matplotlib package [40].

## Results

**Fig. 3:**
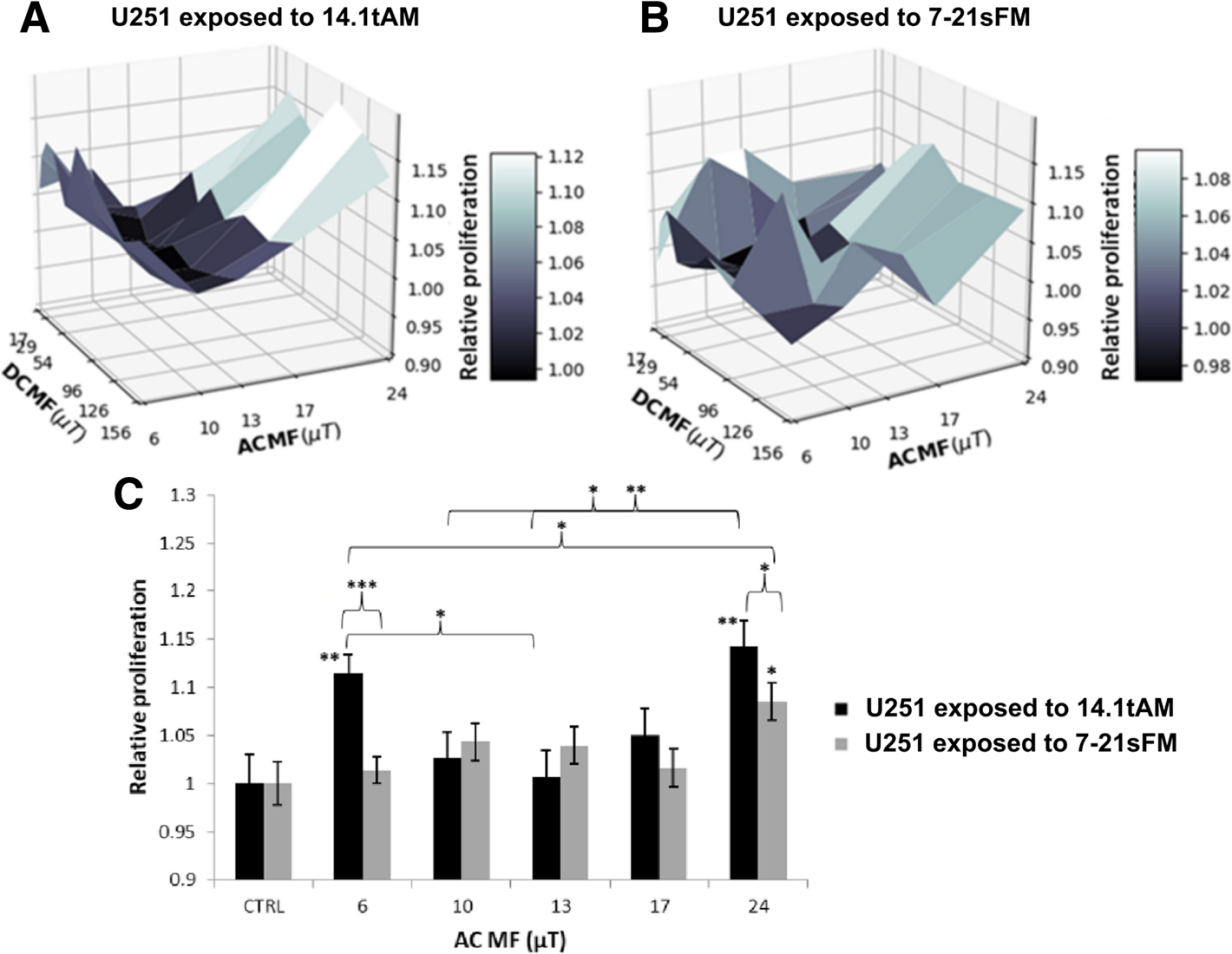
One cell line (U251) exposed to two different signals. **a** Relative proliferation of U251 cells exposed to signals (**a**) 14.1tAM and (**b**) 7-21sFM, for each combination of DC and AC fields. **c** Average over all DC field values: for each AC field, the average and standard error of the mean (SEM) of a total of 30 wells is shown (3 independent experiments, 10 wells (columns 2 through 11) for each AC field, per experiment). Bars are ± SEM for 3 independent experiments. *: *p* < 0.05, **: *p* < 0.01, ***: *p* < 0.001. Relative proliferation of cells treated with 1 nM Calyculin A (as positive control for inhibition) was of 0.008 ± 0.044 (not shown in the plot)

Figures 3a-b show the proliferation of the U251 cultures exposed to signals 14.1tAM and 7-21sFM, relative to control. These 3D plots show only the average of three experiments, while the bar plot of Fig. 3c (see below) is indispensable for a comprehensive interpretation of the data through the statistical analysis. While Fig. 3b seems rather “noisy”, Fig. 3a has a half-tube shape with its axis parallel to the rows of the microplate, i.e., with similar values of proliferation for constant AC MF, regardless of the DC MF. Indeed, we verified with a factorial ANOVA analysis that, within the tested DC MF range (17–156 μT), DC intensity did not introduce any statistically significant effect. However, it is clear that DC fields other than the ones tested (or even zero DC field) could have yielded different results. This, in turn, allowed us to average over all values of the DC MF (Fig. 3c). Of note, all values of AC MF hereinafter in the text and figures are peak values. For the 14.1tAM signal, there was a significant increase for AC MF = 6 μT (11.6%, *p* < 0.01) and for AC MF = 24 μT (14.3%, p < 0.01) as compared against control. For the 7-21sFM signal the only significant difference against control was at AC MF = 24 μT (increase of 8.5%, *p* < 0.05). It is also shown that there were significant differences for the same AC MF intensity but different signals (at AC MF’s 6 μT and 24 μT), and for the same signal but different AC MF’s (6 μT and 13 μT, 10 μT and 24 μT, and 13 μT and 24 μT for 14.1tAM).

**Fig. 4:**
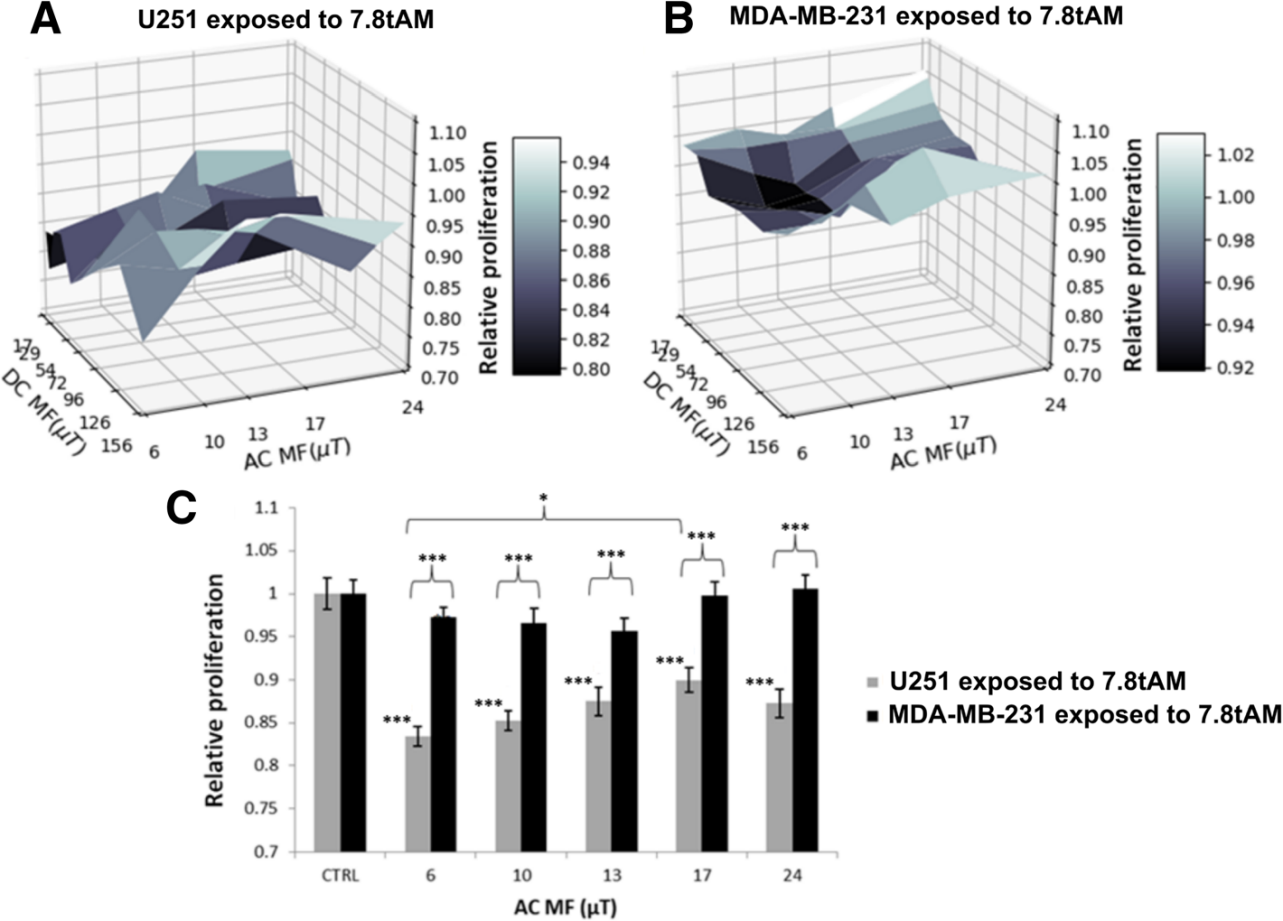
Two cell lines exposed to the same signal. Relative proliferation of (**a**) U251 and (**b**) MDA-MB-231 cells to the 7.8tAM signal, for each combination of DC and AC fields. **c** Average over all DC field values: for each AC field, the average and standard error of the mean (SEM) of a total of 30 wells is shown (3 independent experiments, 10 wells (columns 2 through 11) for each AC field, per experiment). Bars are ± SEM for 3 independent experiments. *: *p* < 0.05, ***: *p* < 0.001. Relative proliferation of U251 cells treated with 1 nM Calyculin A (as positive control for inhibition) was 0.025 ± 0.039, and that of MDA-MB-231 treated with 10 nM was 0.064 ± 0.044 (not shown in the plot)

Since we were interested in a decrease in proliferation of cancer cells, we decided to test a third signal, 7.8tAM, and we did it on two different cell lines (Fig. 4). Again, a factorial ANOVA indicated that in our experiments the DC MF was not an influencing factor, so we averaged data over all DC MF (Fig. 4c). We observed that U251 was responsive for all values of the AC MF, with a maximum decrease in proliferation of 16.6% compared to control (*p* < 0.001), while the MDA-MB-231 was not responsive at all. There were significant differences between the two cell lines for all values of the AC MF (*p* < 0.001) and also, for U251, a small (6.6%), but significant (*p* < 0.05), difference between AC MF 6 μT and 17 μT.

No morphological changes (size and shape) were observed between exposed and controls, or within the exposed plates in any of the experiments.

## Discussion

Many effects of weak ELF-MFs on cell proliferation have already been reported, and both inhibition and stimulation have been observed, depending on cell type and exposure conditions [41, 42]**.** For each biological effect described, the heterogeneity of exposure conditions makes difficult to establish the importance of intensity, frequency and waveform. This study proposes a novel approach to verify the effects of different DC/AC intensities of ELF-MF. The results obtained in this study suggest that, at a given pattern of MF modulation, the screening of the intensity of AC and DC field can be important to find a biological response. For instance, in experiments aiming to elucidate whether 50 Hz power line fields may be carcinogenic, the 50 Hz frequency is naturally a parameter that must be chosen. However, it is not clear if some amplitude-window is present in the biological response to the AC 50 Hz fields, and even less clear what is the relation (if any) between the 50 Hz AC component and the DC field component present at the site. In principle, under the light of what is known so far in magnetobiology, effects could indeed be different for the same frequency and AC amplitude, but different DCs. From what this example illustrates, we sustain that screening is a valuable and, in some cases, unavoidable methodological resource. In sum, we are aware that the need of screening is not universal and does depend on the purpose of the study, but we sustain that it is of importance when looking for the possibility of ELF-MF being carcinogenic (at least for some of the parameters) and also, and perhaps more patently, when looking for a potentially therapeutic use of these fields.

Our results coincide with the literature in that different ELF exposures can elicit various effects in a cell line. For instance, there are reports of bacterial proliferation depending on the field intensity [43–45]. Also, DNA transposition in two strains of *E. coli* was affected by waveform of the ELF-MF, while no differences were seen between different frequencies [46]. Remarkably, we found that the U251 cell line displayed the three possible responses to exposure to Schumann frequencies: no effect, increase, and decrease of proliferation, depending on the MF modulation and amplitude. Under the light of such a behavior, we pose that extreme caution should be put into interpretation of results of single-exposure experiments, e.g., increased proliferation of [47, 48], or the inhibition of anti-apoptotic proteins in [49], malignant cells; or in the search of “therapeutic modulations”, given the concrete possibility of “missing” effects (or even obtaining the opposite ones) that would have been found by sweeping through the exposure parameters. Decrease in cancer cell proliferation is of special interest, since application of ELF-MF is an easily achieved non-invasive method, which may represent a valuable approach in cancer treatment.

The different response of U251 to the signals 14.1tAM and 7.8tAM (with carrier frequencies that could be considered very similar) is reasonably explained if we consider that within virtually all models of interaction, the frequency is a key parameter and, in those that pose resonance-like phenomena, a variation from 14.1 Hz to 7.8 Hz actually represents a rather big difference. For instance, within models based on Larmor precession, the resonance frequency is proportional to the ratio of the charge to the mass of the “engaged” ion. Hence, changing from 14.1 Hz to 7.8 Hz implies a factor (*f*) of 1.81 in the resonance, implying, e.g., that instead of resonating with Mg^2+^ one would be doing so with Na^+^ (*f*_*Mg/Na*_ = 1.89) or instead of with Na^+^, nearly with K^+^ (*f*_*Na/K*_ = 1.70). To affect different ions could trigger completely different effects. Hence, we were not surprised to observe a different outcome (even a change from proliferative to antiproliferative) upon exposure to those two frequencies.

The present study also confirmed that the same exposure can affect differently distinct cell lines. For example, different amount of DNA strand breaks in various human cell lines were observed upon exposure to the same ELF-MF signal [50, 51]. Also, using intermediate intensities, Li et al. [52] reported different effects on the apoptosis of two human hepatosoma cell lines after exposure to the same ELF rotating MFs. Although Simkó [53] proposed that the different redox status of different cell lines could explain the difference of effects upon the same ELF exposure, it is clear that further research into the physical and biochemical mechanisms of interaction of ELF-MF with living cells is still necessary. With regards to our results, of note is that Öskan et al. [54] found that proliferation of U251 and MDA-MB-231 was different upon treatment with a vegetal extract. Although their results were opposite to ours (inhibition occurred for MDA-MB-231, but not for U251), they provide an independent report of a different response upon the same treatment. As to a possible explanation of our observations, Hall et al. [55] reported that the mitochondrial calcium uniporter (MCU) is dispensable for the progression of MDA-MB-231 (since knockdown of MCU did not affect reactive oxygen production or cause significant effects on clonogenic cell survival of cells exposed to irradiation, chemotherapeutic agents, or nutrient deprivation). However, for progression of other cancer cell types - cervical, colon, prostate - they found that the MCU is necessary. While to the best of our knowledge the role of MCU in glioblastoma has not thus far been investigated, we hypothesize that, similarly to cervical, colon, and prostate cancer cell types, glioblastoma could be highly dependent on MCU; if our signal 7.8tAM would affect this ionic channel, only U251 cells would be affected in their growth, whereas MDA-MB-231 cells would be unresponsive. This difference in sensitivity calls the attention to the possible effects of ELF-MF on the mitochondrial activity of cancer cells, a subject that has recently been investigated by Destefanis et al. [56], who found that a 50 Hz 12 μT (rms) MF for 7 days inhibited proliferation of four cancer cell lines, and induced changes in the mitochondrial protein profile.

The aim of this study was to provide a proof of concept of the utility of the system of coils in screening a range of intensities, and this goal was reached by the experiments carried out on U251 cells. In addition, we wondered if the signal that had been effective on U251 would also influence the growth of MDA-MB-231 cells. With the purpose of finding a signal that would inhibit the proliferation of several cancer cell types, we were not interested in testing neither the 7-21sFM (ineffective on U251 cells), nor 14.1tAM (triggering the opposite effect on U251 cells: the increase of proliferation). We found that the MDA-MB-231 cells were insensitive to the tested signal and intensities, reinforcing the principle that each cell type may have a different response to the electromagnetic radiation.

In spite of the increasing number of reports on the subject, it must be noted that at present there is no well-established biophysical rationale for the exact definition of the exposure parameters to be tested as possibly therapeutic, and we have not made a contribution in this respect. Thus, the time modulation and exact AC and DC intensities (and angle) of the signals used in our experiments - as in the great majority of the literature - still comes down to trial and error. An interesting exception to this is the work by Lucia et al. [57], in which the authors do provide a model for predicting effective frequencies of the ELF-MF exposure, and experiments in line with their predictions. While the authors state that according to their model the AC intensity is less important than frequency, our results (and models such as the ones based on Larmor precession, or quantum states interference) indicate otherwise. However, the fact that our results did not depend on DC intensity are in line with Lucia et al.’s model (which does not include DC field as a relevant parameter). Either with relatively arbitrary or rationally designed signals, we present a new tool that can sensibly accelerate the search of effective parameters.

While Cherry [34] proposes that given that brain waves’ bandwidth is in the same range of the SRFs they could “tune-in” to them, we agree with Palmer et al. [58] in that Cherry does not provide with a plausible mechanism for that “tuning”; and that a proper biophysical explanation of it is a key for further pursuing Cherry’s hypothesis. Furthermore, we point out here that, according to the fact that not only frequency-windows, but also amplitude-windows can exist upon exposure to ELF-MF (predicted by theoretical models [29], and shown in experiments [44, 45, 59, 60], and also in this work), such “tuning” (provided it exists) should occur not only at the right frequencies, but also at the right amplitudes. Under the light of such complexity, the fact that we found an effect for brain cancer cells (presumably more prone to “resonate” with SRFs) but not for breast cancer cells should be taken only as a suggestive and circumstantial coincidence, far for representing any kind of confirmation of Cherry’s “tuning” hypothesis. Lastly, our experiments did not show an especially remarkable effect, so they do not support the idea of MFs at SRFs being particularly effective, at least with the modulations and intensities that we studied.

From all of the afore said, we can only conclude that the subject remains extremely complicated, and that careful screening through the different parameters of the exposure (DC amplitude, AC amplitude and frequency, intermittence, duration, etc.) sounds like a desirable (if not unavoidable) path for unravelling the biophysical mechanisms underlying the interaction of ELF-MF with living matter.

## Conclusions

We demonstrated, for the first time, that the system of flatten orthogonal coils used in our experiments was indeed useful for testing multiple field conditions in a single experiment. By using it, we found that proliferation of cancer cells was decreased, increased, or not-affected by weak ELF-MF, in dependence on their amplitude and modulation, and the cell line. Our results are in line with the literature in that different cell lines can respond differently to the same magnetic field exposure, and that the same cell line can respond differently to different modulations/amplitudes of the fields. The use of the orthogonal set of coils constitutes a novel approach for screening in the search for effective ELF-MF parameters for affecting cancer cell growth.

## Additional file

Additional file 1: The file is a Microsoft Excel spreadsheet with the measurements of the magnetic fields utilized in the experiments, and the colorimetric readings from the proliferation assays. (XLSX 56 kb)

## Abbreviations

AC: Alternated current
DC: Direct current
ELF-MF: Extremely low frequency magnetic fields
PMMA: Polymethyl methacrylate
